# Uptake, Translocation, Toxicity, and Impact of Nanoparticles on Plant Physiological Processes

**DOI:** 10.3390/plants13223137

**Published:** 2024-11-07

**Authors:** Maduraimuthu Djanaguiraman, Veerappan Anbazhagan, Om Parkash Dhankher, P. V. Vara Prasad

**Affiliations:** 1 Department of Agronomy, Kansas State University, Manhattan, KS 66506, USA; 2 Department of Chemistry, School of Chemical and Biotechnology, SASTRA Deemed University, Thanjavur 613401, India; anbugv@gmail.com; 3 Stockbridge School of Agriculture, University of Massachusetts, Amherst, MA 01003, USA; parkash@umass.edu; 4 Department of Crop Physiology, Tamil Nadu Agricultural University, Coimbatore 641003, India

**Keywords:** abiotic stress, metabolism, nanoparticles, toxicity, translocation, uptake

## Abstract

The application of nanotechnology in agriculture has increased rapidly. However, the fate and effects of various nanoparticles on the soil, plants, and humans are not fully understood. Reports indicate that nanoparticles exhibit positive and negative impacts on biota due to their size, surface property, concentration within the system, and species or cell type under test. In plants, nanoparticles are translocated either by apoplast or symplast pathway or both. Also, it is not clear whether the nanoparticles entering the plant system remain as nanoparticles or are biotransformed into ionic forms or other organic compounds. Controversial results on the toxicity effects of nanomaterials on the plant system are available. In general, the nanomaterial toxicity was exerted by producing reactive oxygen species, leading to damage or denaturation of various biomolecules. The intensity of cyto- and geno-toxicity depends on the physical and chemical properties of nanoparticles. Based on the literature survey, it is observed that the effects of nanoparticles on the growth, photosynthesis, and primary and secondary metabolism of plants are both positive and negative; the response of these processes to the nanoparticle was associated with the type of nanoparticle, the concentration within the tissue, crop species, and stage of growth. Future studies should focus on addressing the key knowledge gaps in understanding the responses of plants to nanoparticles at all levels through global transcriptome, proteome, and metabolome assays and evaluating nanoparticles under field conditions at realistic exposure concentrations to determine the level of entry of nanoparticles into the food chain and assess the impact of nanoparticles on the ecosystem.

## 1. Introduction

The world population is around 7.8 billion, of which 820 million people are experiencing chronic hunger and 135 million people from 55 countries are living in acute food-insecurity zones. Apart from this, the world population is projected to be around 9.8 billion by 2050, and global agricultural production needs to be doubled or 593 million hectares of land should be brought under cultivation to feed this ever-increasing population. World Resources Institute has identified 22 solutions to close these gaps. The key solutions include reducing food loss and waste, increasing food production without increasing agricultural land, protecting and restoring natural ecosystems, increasing fish availability, and reducing greenhouse gas emissions from agricultural production [1]. To achieve Sustainable Development Goal 2, “zero hunger and improved nutrition”, global food systems need a major transformation. Isolated fixes cannot solve complex issues, and agricultural technologies are important [2].

Producing enough food for the growing population with the currently available crop production technology is challenging due to changes in climatic conditions. Increasing the intensity of agricultural production in a sustainable way is not followed in many parts of the world, leading to the contamination of the environment and exploitation of resources. Historical observations and model simulations have suggested a high risk of drought across the globe [3], and unpredictable rainfall patterns or insufficient rainfall have led to a substantial loss in crop yield. On a global scale, the reduction in yields of cereals, legumes, and oilseeds due to drought stress was 10, 50, and 30%, respectively [4,5,6]. According to the global climate change scenario, average temperatures are expected to increase from 1.8 to 4.0 °C or even higher by the end of this century compared to the 1980–2000 average [7]. Each degree Celsius increase in the average growing season temperature may decrease crop yield by up to 17% [8]. Thus, it is essential to develop crop management strategies to alleviate the effects of drought and high temperatures on crops to obtain economic yields.

Future yield increases are essential to meet the demand. The conventional breeding approach may be an option to achieve genetic gain; however, it is time-consuming. In contrast, new advances in molecular biology and nanotechnology offer great promise for attaining yield gains. There is no doubt that sustainable agricultural growth is possible with the application of nanotechnology. Nanotechnology deals with the manipulation of matter on an atomic, molecular, or supramolecular level. The application of nanoparticles is of great scientific interest due to the diverse applications of nanotechnology in agriculture. A nanometer is one-billionth of a meter, and nano refers to a scale between 1 and 100 nm in at least one dimension. Hence, nanomaterials can be zero-dimensional (all three dimensions are at the nanoscale; for example, nanoparticles), one-dimensional (one dimension in the nanoscale and the other two in the macroscale; for example, nanofibers and nanowires), and two-dimensional (two dimensions in the nanoscale and the others in the macroscale; for example, nanosheets and thin films) [9]. Nanomaterials show unique properties at the nanoscale compared to their bulk counterparts because of the increased surface area and quantum confinement. Nanomaterials possess high surface-to-volume ratios, which makes them interact with the environment more intensely than their bulk counterpart.

Based on origin, nanoparticles have three types, namely natural, incidental, and engineered nanoparticles. Natural nanoparticles exist on earth due to volcanic dust, lunar dust, terrestrial dust storms, mineral composites, photochemical reactions, forest fires, simple erosion, etc. Incidental nanoparticles are formed by human-made industrial processes like petrol/diesel exhaust, coal combustion, welding fumes, etc. Engineered nanoparticles are categorized into five types, namely carbon-based nanoparticles, metal-based nanoparticles, magnetic nanoparticles, dendrimers, and composites. In general, nanomaterials are synthesized by physical, chemical, biological, and hybrid methods. The physical method involves mechanical and vapor deposition methods. Examples of mechanical methods include high-energy ball milling and melt mixing. Similarly, examples of vapor deposition comprise physical vapor deposition, sputter deposition, and electric arc methods. Techniques like sol–gel, Langmuir–Blodgett film, and inverse micelles are examples of chemical methods. Regarding biological methods, biomembranes, deoxyribonucleic acid, enzymes, and microorganisms are used to synthesize nanomaterials. In the hybrid method, electrochemical, chemical vapor deposition, and microemulsion are commonly used. Detailed information about nanomaterials’ dimensions, properties, types, and synthesis are already available [10]. In the agriculture sector, nanotechnology is likely to facilitate the delivery of pesticides, fertilizers, and hormones in a controlled manner, nano-sensors for monitoring soil and plant health, nanochips for tracking food safety, the development of genetically modified crops, and precision farming techniques.

This review summarizes the current literature on the bioaccumulation, uptake, translocation, and toxicity of nanoparticles to plants and their impacts on key physiological processes.

## 2. Bioaccumulation and Modes of Exposure of Nanoparticles

**Figure 1:**
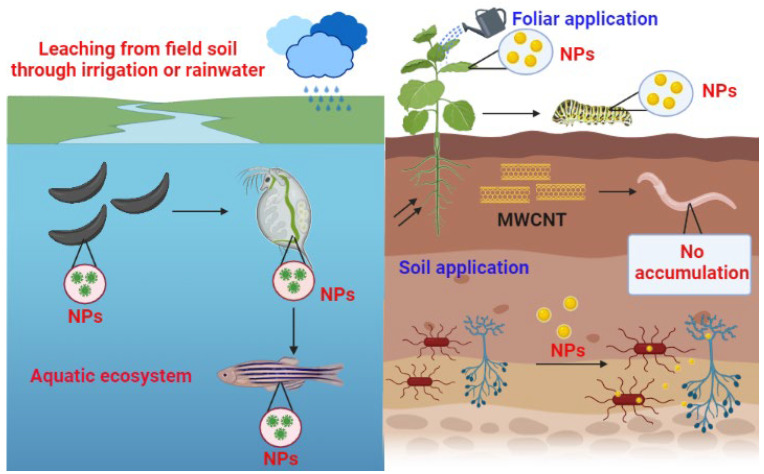
A representative diagram showing the bioaccumulation of nanoparticles on the aquatic and soil ecosystem.

Terrestrial plants are directly exposed to nanoparticles through the soil, water, and air. The absorption of nanoparticles by plants, their transport within the system, and bioaccumulation through the food chain are plausible. Comprehensive reviews of nanotoxicity in humans, small mammals, invertebrates, and aquatic organisms have been published [11,12,13]. However, studies related to nanotoxicity to terrestrial and aquatic plants are limited [14,15,16]. Bioaccumulation is a concept that explains the process associated with the transfer of nanoparticles from low to superior trophic levels (Figure 1).

Aquatic flora constitutes the elementary level of the food chain because they are the primary producers of biomass. The exposure of algae to nanoparticles at a potentially toxic level may affect the natural ecosystems by accumulating *Daphnia magna* (a typical water flea) through algae, thereby incorporating them into the food chains [17]. A possible adverse effect of nanoparticles on algae has severe consequences on the entire aquatic food web. Therefore, it is essential to identify the mechanism of nanotoxicity or internalization in an aquatic species to quantify the risk assessment [18]. The transmission or enrichment of nanoparticles through the aquatic food chain may lead to toxic effects on high trophic-level organisms in the food chain. The report indicated that quantum dots could be transported along the food chain from green algae (*Pseudokirchneriella subcapitata* L.) to water fleas (*Ceriodaphnia dubia* L.). Marine organisms accumulate nanoparticles in either the gills, gut, digestive glands, or digestive tract and affect the growth and reproduction of *Daphnia magna* [19]. For example, the trophic transfer of SiO_2_ and CeO_2_ nanoparticles from *Cricosphaera elongata* to the larvae of *Paracentrotus lividus* has shown that the survival rate of larvae fed with *Cricosphaera elongata* was significantly reduced and had abnormal developments like skeletal degeneration and altered rudiment growth [20]. It has also been observed that nanoparticle transfer from green algae to fish had a more significant impact on fish behavior [21]. The above findings demonstrated that nanoparticles from lower trophic levels could be transported through the food chain and accumulated in higher trophic level organisms. The nanoparticles can penetrate the tissue barrier, and then they can accumulate in the liver, kidney, spleen, muscle, stomach, and intestine of organisms of higher trophic levels [22]. Overall, it is clear that nanoparticles may cause a risk to human beings if they are not tested for their toxicity potential. Before using any nanoparticle in aquatic, soil, or terrestrial ecosystems, it must be evaluated for toxicity at all trophic levels.

The primary methods of nanoparticle application to plants include direct seed, seedling, or grown-up plant exposure through in vitro culture media or soil, spraying, hydroponics, isolated cells, protoplast incubation, and biolistics. The primary nanoparticle application method for seeds is directly soaking the seeds in a nanomaterial suspension or germinating in nanoparticle-spiked growth media or soil. Nanoparticle exposure through the roots will facilitate nanoparticle translocation from the roots to the shoots through the xylem tissue, provided it is mobile. However, small-sized nanoparticles with large surface areas tend to aggregate in aqueous media, which modulates their bioavailability. Apart from this, root tips and hairs secrete a significant amount of mucilage composed of hydrated polysaccharides, which might contribute to the aggregation of nanoparticles and clogging of cell membrane pores, leading to the restricted entry of nanoparticles into plant systems [23]. The hydroponics method is extensively used for semiaquatic and terrestrial plants for its growth by immersing the roots in the nutrient solution or an inert medium like perlite, gravel, or turface [24,25]. Through this method, the effect of soil microorganisms and interactions of nanomaterials and nutrients cannot be quantified with more precision.

The plant cell walls are semipermeable, and the average pore size of the plant cell wall is less than several nanometers. Therefore, any foreign materials with sizes greater than the pore diameter would be restricted from entering the active plant cell. However, it has been shown that nanoparticles may also penetrate plant cells by going through or bypassing the cell wall by creating new channels. Currently, most studies on plants involve the exposure of nanomaterials through foliage as a foliar spray to mimic the interaction of nanoparticles present in the atmosphere with leaves or the application of purpose-made nanoparticles. Nanoparticles applied to leaf surfaces gain entry through stomatal openings or the bases of trichomes and are subsequently translocated to various tissues [14,26,27].

## 3. Uptake and Translocation of Nanomaterials

Terrestrial and aquatic plants are expected to be exposed to nanomaterials from sewage sludges, pesticides, and fertilizers in nanoformulations, wastewater treatment effluent, and atmospheric sources from factory or laboratory exhaust, including contaminated rainfall containing nanomaterials [28]. Submerged hydrophytes exhibit much faster mass transfer rates for the uptake of CO_2_ and other dissolved gases from water, and this mass transfer is facilitated by the underdeveloped or absent epidermis and/or protective lipophilic cuticle [29]. Consequently, nanoparticles well dispersed in water can directly interact with the cellulose present in the cell wall, facilitating nanoparticle uptake. In terrestrial plants, the absorption and mobility of nanomaterials are found to be low [28], and their mobility in soil was found to be even lower [30]. Environmental conditions, mucilage, root exudates, hyphae, and bacteria populations can also significantly alter nanoparticle mobility in soil by slow desorption kinetics or sequestration [31,32].

Microorganisms associated with plants create symbiotic microenvironments and soil aggregates or biomineralized products [33]. The symbiotic microenvironment affects metal and metal oxide nanoparticle mobility and uptake in plants. Mycorrhizal fungi form a symbiotic relationship with the majority of higher plants, and this association increases the active root surface by ten-fold, leading to improved water, nutrients, and likely nanoparticles from the soil [34]. However, a study on clover (*Trifolium repens* L.) has indicated that arbuscular mycorrhizal fungi increased the soil porosity near nano-silver (Ag)- and nano-iron (Fe_2_O_3_)-exposed roots and reduced the nano-Ag uptake [35]. Mucilage and exudates are excreted into the rhizosphere via the root cap (border cells) and root hairs of plants. Insoluble, high-molecular-weight mucilage and soluble, low-molecular-weight exudates are involved in sensing, protecting, and lytic agents of the cell surface and are the primary barriers for nanoparticle entry into the root system. The nanoparticles present in soil interact with mucilage and exudates and accumulate on the outer mucilaginous plant surface [36,37,38]. Little is known about the role of mucilages or exudates on nanoparticle uptake into roots. In terrestrial plants, nanoparticles accumulate and aggregate near the root tips, especially the root cap and hairs [37,38,39,40]. The mucilage and exudates acidify the rhizosphere, leading to the dissolution of nanoparticles [36,38,41]. For example, Ce^3+^ from CeO_2_ contributed significantly to the uptake of total cerium in pumpkin (*Cucurbita maxima* L.), wheat (*Triticum aestivum* L.), and sunflower (*Helianthus annus* L.) [42]. Nanoparticles with positive surface charges are associated with accumulation on the root surface due to mucilage, which reduces the mobility of nanoparticles from root to shoot, validated by using positively and negatively charged nano-Au particles [43]. However, some superparamagnetic Fe_2_O_3_ nanoparticles are transported from roots to shoots regardless of charge [44]. Further, negatively charged nanoparticles such as Alizarin Red S-labeled nano-TiO_2_ can strongly accumulate in the thick mucilage sheath of seeds [45,46].

The cuticle protects the plants from uncontrolled water loss and entry of pathogens by affecting their attachment to the leaves. The hydrophobic nature and chemical inertness of the cuticle repel the polar compound from entering the plant system and act as a barrier for nanoparticles to enter through aerial parts. Studies have shown that negatively charged or neutral nanoparticles were accumulated in the cuticle [47,48]. However, the study also showed that nanoparticles like TiO_2_ cause holes in the cuticle and enter the plant system [49]. Plant species with epicuticular waxes provide additional protection for nanoparticle entry through the aerial parts. Like pesticides, nanoparticle uptake can be increased by using a surfactant [48]. Dicot cuticles are more permeable than monocots for lipid-based nanoformulations due to their altered wax morphology [48].

A polar pathway has been proposed for hydrophilic compounds, mainly through thin permeable regions of the cuticle near the cell wall or cuticle present near the cell junction [50]. The cellulose in the cell wall is the next barrier for nanoparticle entry into the plant system. The cell wall thickness is a few hundred nanometers up to several µm with a uniform thickness. However, the endodermis and epidermis aged secondary cell wall thickness will still be higher, making it tough to enter. Suberin, CaCO_3_, MgCO_3_, and SiO_2_ are present in the endo- and epi-dermis, which can potentially affect the nanoparticle transport [34]. Nanoparticle uptake across the plant cell wall primarily depends on its size and pore size. The dynamic pore size contradicts the current assumption that nanoparticles crossing the cell wall are limited to diameters of 5–20 nm [51,52]. Internalized and translocating nanoparticles with primary particle diameters up to ~50 nm were present in the cytoplasm [53]. Kim et al. [54] showed that nanoparticles like nano zerovalent iron (ZVI) degrade the pectins present in the cell wall through hydroxyl radical reaction, leading to increased mobility in the plant tissues. The plant plasma membrane is selectively permeable. Therefore, polar molecules like water and ions cannot easily diffuse through the phospholipid bilayer, and these molecules are translocated through the apoplast.

**Figure 2:**
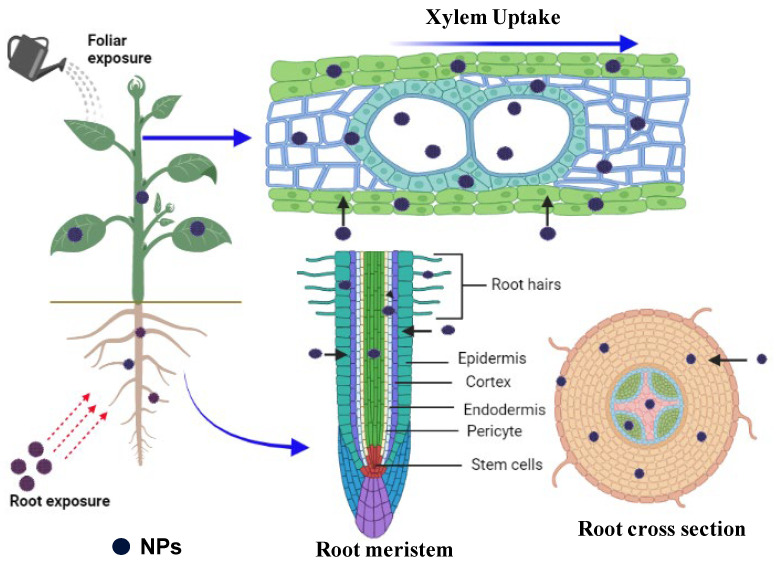
Representative diagram showing nanoparticle uptake and translocation in plants.

Hydathodes, small cavities at the tip of leaves lacking cuticles, are another mode of entry of nanoparticles in plants. The uptake and excretion of nanoparticles through hydathodes have been reported by Hong et al. [55]. However, the accumulation of nanoparticles near hydathodes was also found in cucumbers (*Cucumis sativus* L.). The major entry point of nanoparticles in plants is through the stomata. During the daytime, plants tend to keep their stomata open for gas exchange, showing increased permeability for polar molecules [50]. The foliar uptake of nanoparticles has resulted in the translocation of nanoparticles from stomatal cavities to plant tissues, namely adjacent cells, vascular tissues, and roots [56]. The translocation of nanoparticles coincided with its solubility nature. Direct observations of stomatal uptake of non-dissolving nanoparticles and translocation from stomatal cavities to adjacent tissues are rarely reported. Blockages of stomatal pores and vascular tissues by foliar spray of nanoparticles leading to decreased water fluxes have been reported by Lu et al. [57] (Figure 2).

Nanoparticles can enter terrestrial plants through roots or shoots, and for uptake and translocation, nanoparticles undergo various chemical and physiological barriers. Currently, cellular penetration is the most accepted mode of entry through which nanoparticles interact with plants [58,59]. The size of the nanoparticle is the primary determinant for penetration into plant tissues. The plant cell wall acts as a barrier to the entry of nanoparticles into plant cells because the pore diameter in the cell wall ranges from 5 to 20 nm, which significantly changes with plant species [60]. Therefore, nanoparticles or aggregates of nanoparticles that fall within the above size range could quickly pass through the cell wall and reach the plasma membrane [61]. Other studies have also shown that a 40–50 nm nanoparticle dimension could move and accumulate inside the cells [25,62]. Nanoparticles adhered to the cell wall may move through endocytosis, and further, they may travel to different plant tissues by symplast transport [51]. Plasmodesmata strongly regulate the symplastic transport of nanoparticles. The presence of nanoparticles or precipitate in or near plasmodesmata is the first evidence to suggest that nanoparticles of <15–40 nm in diameter can enter plasmodesmata [63]. However, the rare direct observation of nanoparticles in plasmodesmata indicates that the symplastic movement of the nanoparticles is still unknown. Nanoparticles are most often detected in the apoplast and not in the symplast. Studies reporting the movement of nanoparticles through the symplast are based on the size of the nanoparticle, radical nanoparticle transport in plants, root–shoot translocation, the accumulation of nanoparticles in different tissues, and the presence of nanoparticles in the xylem and phloem [44,64,65].

Wong et al. [66] proposed a mathematical model, namely the lipid exchange envelope and penetration model (LEEP). This model assumes interactions between charged nanoparticles and the surface charges on the chloroplast membrane to soften the membrane, causing an expanded and fluid state. This process is expected to be favored thermodynamically and allows the nanoparticles to penetrate the chloroplast. Glycerolipids, namely galactolipids and sulfolipids, comprise 52% of the total lipids in the outer chloroplast membranes and are then considered to chemically interact with the charged nanoparticles as they are transported across the chloroplast membrane. The lipid-coated nanoparticles then diffuse into the chloroplast before being trapped irreversibly in the interior. Kinetically trapped nanoparticles are unable to be transported out of the chloroplast, and the chloroplast membrane reheals. However, the LEEP model does not predict the efficiency of nanoparticle entry into the chloroplast and assumes the nanoparticles are spherical. The composition of lipids, ligand density on the nanoparticles, and incubation time are also not considered in the LEEP model. The study stated that size, magnitude, and zeta potentials are the key players in determining nanoparticle transport inside the plant. Studies have also indicated the chance of enlarging pores or forming new cell wall pores by nanoparticles via interaction with the cell wall, leading to enhanced uptake and translocation. The type and chemical composition of nanoparticles also influences the uptake [67], whereas morphology has also been demonstrated to be a determinant in cellular uptake [68]. Surface modification and functionalization of the nanoparticle can change the properties, leading to enhanced absorption and accumulation by the plants [69]. Plant species differ significantly in their physiology, leading to changes in the uptake of nanoparticles, and the method of application is also a significant determinant in plant uptake, translocation, and the accumulation of nanoparticles.

Zhu et al. [70] unambiguously showed that pumpkin (*Cucurbita maxima* L.) roots took Fe_2_O_3_ nanoparticles and translocated them through plant tissues. This study showed that 45% of the fed Fe_2_O_3_ nanoparticles accumulated in the roots, and less than 1% were detected in the leaves. However, there was no uptake and transport of Fe_2_O_3_ nanoparticles in lima beans (*Phaseolus limensis* L.). In another study, Lin et al. [71] showed that fullerene (C_70_) was readily taken up by the roots and transported to the shoots, and they also found that C_70_ could be transported from the leaves to the roots through phloem if it was applied through foliar mode. However, similar results were not observed with multi-walled carbon nanotubes (MWCNTs), which could be associated with the size. Research on the effect of zinc oxide (ZnO) nanoparticles on ryegrass (*Lolium perenne* L.) has shown no upward translocation of ZnO nanoparticles from roots to shoots [72]. In contrast, nanoselenium (Se^0^) moved from the roots to shoots in sorghum (*Sorghum bicolor* L. Moench; [25]). This study was conducted in liquid media. However, the response of plants in soil was not known.

Wang et al. [37] confirmed the presence of CuO nanoparticles in maize (*Zea mays* L.) roots exposed to 100 mg L^−1^ of CuO nanoparticles. CuO was present inside the cell wall of the epidermis, intercellular space, cytoplasm of cortical cells, and nuclei. The presence of CuO nanoparticles in the intercellular space demonstrates that nanoparticles may pass through the epidermis and cortex via apoplastic pathways. Also, the authors observed the presence of endosomes upon treatment with CuO nanoparticles, suggesting that endocytosis may be one mode of CuO nanoparticle entry into plant cells. Once nanoparticles have entered the plant’s system, further transport takes place through the vascular system of the phloem [73]. The pressure gradient of photosynthate in leaves drives a flowing stream of nanoparticles and assists them in moving through the phloem-loading mechanism. This pathway of nanoparticle transport through the xylem and phloem has also been verified previously [68,73].

## 4. Toxicity of Nanoparticles

Most of the studies explaining nanoparticle toxicity in the plant system have concentrated on seed germination and biomass accumulation [74]. Seed germination, plant height, leaf area, and biomass accumulation are the best morphological traits to assess the phytotoxic effect of nanoparticles in plants [75]. The cytotoxic effects can be quantified through enzyme activity, pollen development, photosynthesis, reactive oxygen species (ROS) production, and nucleic acid integrity [76,77]. Nevertheless, the response of plants to nanoparticles differs with the species and type of nanoparticles [14]. The most common nanoparticle toxicity mechanisms in plants include the dissolution and release of toxic ions, size- and shape-dependent damage and clogging, redox cycling and the Fenton reaction, binding interactions with biomolecules, and oxidation and surface catalytic reactions [78]. The above-proposed mechanism was based on the following findings: clogging of pores and barriers in the apoplastic stream [79], reduction in photosynthetic processes through ROS generation [80], and damage in DNA structures [77,79]. The nanoparticles induce various morphological, physiological, and anatomical changes in plants, depending on their size, chemical composition, shape and angle of curvature, chemical structure, and surface property [78,81]. From the toxicological perspective, particle size and surface area are critical because the decrease in particle size increases the surface area and the proportion of atoms or molecules on the surface layer of the particle [82]. These size-dependent properties influence the reactivity and the ability to cross the physiological barrier. However, it is not clear that the variation in toxicity results from changes in size-dependent specific surface area. For example, for a given mass, 12.5 nm SiO_2_ nanoparticles are more toxic to green algae (*Pseudokirchneriella subcapitata* L.) than 27.0 nm nanoparticles. However, when the concentration was expressed on a surface area basis, no significant difference in toxicity was observed between the sizes, indicating that nanotoxicity is related to surface area and not to mass [83].

Apart from the surface area, the shape of nanoparticles also influences toxicity and uptake. For instance, anatase TiO_2_ nanoparticles are more biologically active than rutile TiO_2_, as evidenced by the induction of cell necrosis and membrane leakage by anatase nanoparticles, while rutile nanoparticles induce apoptosis through ROS formation [84]. Similarly, ZnO nanopyramids showed very strong inhibition of the enzyme β-galactosidase compared to ZnO nanoplates and spheres [85]. Apart from intrinsic properties, extrinsic properties like surface charge (zeta potential) and coating and stability characteristics like dissolution, particle aggregation, and valence state on the surface are also critical in the uptake and translocation of nanoparticles. These extrinsic properties are influenced by suspending media ionic strength, pH, and the presence of organic molecules or surfactants. Studies have shown the direct effects of nanoparticles [86]; however, no nano-specific effect of ZnO nanoparticles was observed in plants when ZnO was applied to soil [87]. Nanoparticles that have a positive charge on the surface are more likely to be absorbed through the induction of endocytosis across the cell membrane [88], whereas nanoparticles with a negatively charged coating are more likely to be transported through vascular tissues [88]. Therefore, the behavior of nanoparticles in plants can be altered through surface modification or surface coating [89].

**Figure 3:**
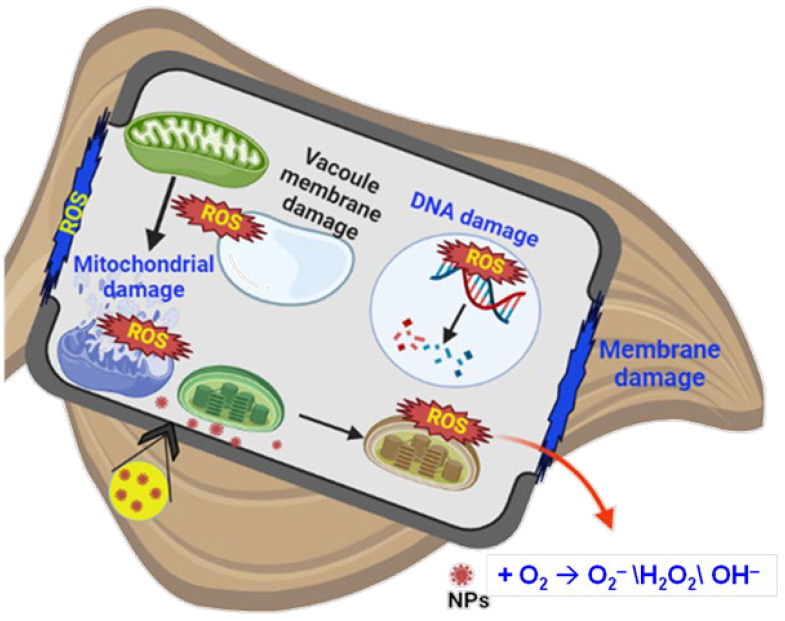
Representative diagram depicting the oxidative damage caused by nanoparticles.

Moreover, the surface coating may reduce the dissolution and release of toxic ions. Reports have shown both positive and phytotoxic effects of nanoparticles on plants. For example, carbon nanotubes (CNTs) facilitated seed germination by penetrating the seed coat, leading to increased water uptake in seeds of Bog birch (*Betula pumila* L.) and Labrador tea (*Rhododendron groenlandicum* L.) [90] and rice (*Oryza sativa* L.; [91]). However, CNT inhibited the root growth of tomatoes and lettuce (*Lactuca sativa* L.; [92]). Likewise, TiO_2_ nanoparticles increased the photosynthetic rate in spinach (*Spinacia oleracea* L.; [93]) and also caused oxidative stress (Figure 3).

Nanoparticles may interfere with plant metabolism, namely micronutrient uptake [94], oxidative processes [95], and gene regulation [96]. It has been reported that if the nanoparticles are present in excess, they cause ROS production, resulting in an oxidative burst [97]. Nanoparticles that enter the plant cells interfere with the electron transport chain of mitochondria and chloroplast, resulting in an oxidative burst through the increased production of ROS [98]. The ROS formed by the interaction of nanoparticles with cellular components can modify the protein structure, induce lipid peroxidation, and damage nucleic acids [99,100]. In addition to this, the ROS that form can either induce apoptosis or necrosis, leading to cell death [98]. However, ROS formed by nanoparticles are also involved in the signaling process, which leads to stress tolerance. Based on the concentration of ROS in a plant cell, the role of ROS (signaling or damaging) is explained. The plant cell has a well-structured antioxidant defense system to control the level of ROS, which includes enzymatic and non-enzymatic molecules. The primary antioxidant enzymes involved in the scavenging of ROS are superoxide dismutase (SOD), catalase (CAT), peroxidase (POX), ascorbate peroxidase (APX), and glutathione peroxidase (GPX), and non-enzymatic scavenging occurs through ascorbate, glutathione, carotenoids, tocopherols, and phenols [101]. Ma et al. [14] showed direct evidence of the role of glutathione in detoxifying the negative effect of Ag NPs. Reports have demonstrated increased production of antioxidant enzymes upon exposure to nanoparticles [102,103], which indicates the regulation of the antioxidant defense system by nanoparticles in plants. Apart from the antioxidant defense system, nanoparticles are also involved in regulating hormones produced in plants [104]. Based on this, it is clear that nanomaterials are involved in the expression of genes associated with antioxidant defense and hormone biosynthesis.

Photosynthesis is a good measure of the overall performance of the plant, and thus, the measurement of photosynthetic pigments and the photosynthetic rate is a good indicator for assessing nanoparticle toxicity in plants. Giraldo et al. [105] reported that embedded single-walled carbon nanotubes in the isolated chloroplast increased the photosynthetic activity by three-fold compared to its controls through enhanced electron transport rates. Similarly, SiO_2_ nanoparticles improved the photosynthetic rate by improving carbonic anhydrase activity and the synthesis of photosynthetic pigments [106]. Nano-anatase improved photosynthetic carbon assimilation by activating the Rubisco (Ribulose-1,5-bisphosphate carboxylase/oxygenase) enzyme through the activation of the Rubisco activase enzyme, resulting in the improvement of the Rubisco carboxylation and a high rate of photosynthesis [107]. However, Vishwakarma et al. [108] observed a significant decrease in chlorophyll content, photosystem (PS) II efficiency, and photochemical quenching upon exposure to Ag nanoparticles in *Brassica* sp. Similar to this observation, Nhan et al. [109] observed damaged chloroplast and vascular tissues upon exposure to CeO_2_ nanoparticles. Hence, it can be concluded that different nanoparticles had different toxicity mechanisms, and toxicity is associated with their physical and chemical properties and the type of crop species.

## 5. Nanoparticles and Plant Growth

Plant growth and development start with the seed germination process, followed by the elongation of the root and shoot. Studies have shown that a low concentration of nano-SiO_2_ improved seed germination in maize by enhancing the nutrient availability in the germination medium [110]. Similarly, a low concentration of ZnO nanoparticles enhanced the seed germination in peanuts (*Arachis hypogaea* L.) and wheat (*Triticum aestivum* L.). However, the seed germination process was inhibited at higher concentrations of ZnO nanoparticles [111,112]. Similar to SiO_2_ and ZnO, TiO_2_ nanoparticles enhanced seed germination and promoted canola (*Brassica napus* L.) seedlings [113]. In addition, it was observed that TiO_2_ nanoparticles enhanced the nitrogen metabolism enzymes activity such as nitrate reductase (NRase), glutamate dehydrogenase (GDH), glutamine synthase (GS), and glutamic-pyruvic transaminase, leading to the enhanced conversion of inorganic nitrogen to organic nitrogen [93,114]. Studies have also shown that TiO_2_ nanoparticles improved the Rubisco, SOD, CAT, and POX enzymes’ activity [115]. It has been shown that TiO_2_ nanoparticles counteracted the toxicity of tetracycline antibiotics in *Arabidopsis* and improved plant growth and biomass when co-exposed to TiO_2_ nanoparticles and tetracycline [116].

The addition of multiwalled carbon nanotubes (MWCNTs) to the growth medium stimulated seed germination in barley (*Hordeum vulgare* L.), soybean, and maize. The MWCNTs were detected inside the seed coats using Raman spectroscopy, and transmission electron microscopy indicated that MWCNTs penetrated the seed coat [117]. The authors also reported that genes encoding water channel proteins in soybean, corn, and barley seed coat are regulated by MWCNTs. Thus, many researchers have confirmed the positive effect of CNTs on the seed germination process [81,91]. Nevertheless, it has also been reported that MWCNTs do not positively influence seed germination at low and high concentrations [118].

Studies have shown enhanced stem and root growth of soybean and wheat plants exposed to ZnO nanoparticles compared to the chelated bulk ZnSO_4_-exposed plant [119,120]. Similarly, the growth and biomass of alfalfa (*Medicago sativa* L.), tomato, and cucumber plants [121] sprayed with ZnO was higher than bulk ZnSO_4_-sprayed plants. In pearl millet [*Pennisetum glaucum* (L.) R. Br.], foliar spray of ZnO nanoparticles increased the chlorophyll and carotenoid concentration compared to its control [122]. In cluster beans (*Cyamopsis tetragonoloba* L. Taub.), ZnO nanoparticle treatment increased the shoot length (22.7%), root length (43.4%), total protein (17.2%), chlorophyll concentration (54.5%), and rhizosphere microbial population (13.6%) compared to the bulk ZnO-treated plants [123]. The lower levels of ZnO nanoparticles were not harmful to cell division and early seedling growth of onion and increased root growth and dry weight [124]. In cotton (*Gossypium hirsutum* L.), ZnO nanoparticle application significantly improved the growth rate, biomass, photosynthetic pigment levels, protein content, and activity of antioxidant enzymes compared to control [125]. In contrast, Zhao et al. [126] observed that ZnO nanoparticles had no impact on the growth, gas exchange, and chlorophyll content of cucumber. There are reports on the adverse effects of a high concentration of Zn in a plant system. For example, a high concentration of ZnO nanoparticles induced oxidative stress in a tomato plant, leading to reduced plant growth and biomass accumulation [127].

Experiments on the effects of Ag nanoparticles have shown that Ag nanoparticles penetrated the cell wall and subsequently damaged the cell morphology [128]. In kiwi (*Actinidia* sp.), the application of Ag nanoparticles caused pollen mortality by damaging pollen membranes [129]. Wang et al. [130] reported that higher concentrations of Ag nanoparticle-treated *A. thaliana* plants reduced shoot and root lengths by directly destroying meristematic cells in the root apical meristem. In another study, Nair and Chung [131] showed that the exposure of mung beans (*Vigna radiata* L. R. Wilczek) to Ag nanoparticles caused ROS generation and lipid peroxidation, indicating cellular damage. Although studies have demonstrated both beneficial and harmful impacts of Ag nanoparticles, there is a need to understand the toxicity of Ag nanoparticles at the cellular level in field crops.

TiO_2_ nanoparticles stimulated seed germination, radicle, and plumule growth. The application of TiO_2_ nanoparticles at an appropriate concentration increased the carbon assimilation rate through increased chlorophyll *a*, *b*, and total chlorophyll, carotenoids, and anthocyanins content [132,133]. Li et al. [134] studied the phytotoxicity of TiO_2_ nanoparticles on rapeseed (*Brassica napus* L.), and the result showed that from 500 mg L^−1^ to 4000 mg L^−1^, the activities of SOD, CAT, and POX were increased compared to the control. The application of TiO_2_ nanoparticles significantly improved the morphological and physiological parameters, namely root length, plant height, fresh biomass, photosynthetic gas exchange, chlorophyll content, and nitrate reductase enzyme (NRase) activity, indicating that up to 4000 mg L^−1^ can be used for foliar application.

Zhang et al. [135] reported that ionic cerium, bulk CeO_2_, and CeO_2_ nanoparticles at a 10 mg L^−1^ concentration exhibited negative, positive, and no significant effects, respectively, on radish (*Raphanus raphanistrum* subsp. *sativus* L. Domain) growth. In another study, three different types of CeO_2_ nanoparticles (lab-synthesized 7 nm and 25 nm and commercial CeO_2_ nanoparticles) were evaluated on the growth of *Latuca* species. It was observed that all three CeO_2_ nanoparticles were toxic to three kinds of *Lactuca* species, and different CeO_2_ nanoparticles showed varying degrees of toxicity [136]. Ma et al. [137] reported differential responses of rape mustard (*Brassica napus* L.) plants to CeO_2_ nanoparticles (~250 nm in size) and bulk CeO_2_ particles (~2000 nm in size). The bulk CeO_2_ at 10 and 100 mg L^−1^ enhanced plant biomass by 28% and 35%, respectively, while CeO_2_ nanoparticles at equivalent concentrations did not. The CeO_2_ nanoparticles induced higher H_2_O_2_ content at the whole plant level during the reproductive stage. However, the bulk CeO_2_ particles increased the H_2_O_2_ content at the whole plant level at the vegetative stage. The results indicate that plant responses to CeO_2_ exposure varied with particle size and growth stages. In addition to this, different shapes, uptake, accumulation patterns, and concentrations of Ce^3+^ on the surface of the CeO_2_ nanoparticle and bulk CeO may contribute to the different impacts of bulk and nanosized CeO_2_. Rico et al. [138] showed that CeO_2_ nanoparticle application to rice seedlings altered the antioxidant defense system of the root. At low concentrations (62.5 mg L^−1^), CeO_2_ nanoparticles inhibited H_2_O_2_ production by 75%; however, at 125 and 500 mg L^−1^, the root H_2_O_2_ content was significantly increased, leading to lipid peroxidation and electrolyte leakage. Research has indicated that both antioxidant enzyme activity and oxidant content are dependent on tissue CeO_2_ nanoparticle concentrations.

## 6. Nanoparticles and Physiological Processes

Despite considerable advances in the use of nanoparticles in agriculture, the possible mechanism of action of nanoparticles at the plant physiological or process level is limited.

### 6.1. Photosynthesis

The chloroplast is the site where complex photosynthetic reactions occur, including light and dark reactions. Research has indicated that nanoparticles have both positive and negative effects on the photosynthetic pigment concentration, photochemical efficiency, and quantum yield of PS II depending on the concentration of nanoparticles within the plant and the type of plant species. The major adverse effects include a reduction in chlorophyll and carotenoid content, PSII quantum yield, photophosphorylation, and enzymes of dark reactions of photosynthesis [139,140,141,142,143]. It is acknowledged that the inhibitory effects of nanoparticles on photosynthesis could be associated with oxidative stress, membrane lipid peroxidation, and chloroplast damage. Among the pigments, chlorophyll *a* is more sensitive to photodegradation. Therefore, chlorophyll *a* could be a useful and reliable biomarker for assessing nanomaterial toxicity compared to growth attributes [144]. For example, the application of CeO_2_ nanoparticles caused no obvious symptoms of toxicity but severely decreased the chlorophyll *a* content in rice [145]. Similarly, magnetite (Fe_3_O_4_) nanoparticles and cobalt ferrite (CoFe_2_O_4_) did not show any visible toxicity symptoms in sunflowers but significantly decreased the chlorophyll *a* concentration, relative to the control, by 50% in nano-Fe_3_O_4_ and 28% in nano-CoFe_2_O_4_ treatments [146]. In another study, a suitable indicator for graphene oxide (GO) nanosheet toxicity in microalgae was found to be the chlorophyll *a* concentration because the reduction in chlorophyll *a* concentration happens before the apparent damage to the chloroplast structure [147].

Studies also showed the positive effects of nanoparticles on photosynthesis and photosynthetic pigment concentration. For example, seed treatment with nano-TiO_2_ in tomatoes resulted in seedlings with higher photosynthetic rates and chlorophyll concentrations than untreated control [148]. In another study, TiO_2_ nanoparticles quenched the ROS produced during the photosynthesis process, consequently improving the photosynthetic rate [149]. Furthermore, the application of nano-TiO_2_ to isolated chloroplasts from spinach resulted in an enhanced oxygen evolution rate that was higher than the untreated control and bulk TiO_2._ The enhanced oxygen evolution rate in spinach plants that received TiO_2_ nanoparticles was attributed to the increased light absorption and photochemical reaction activity, which is consistent with the improvement of chlorophyll synthesis and sensitization of TiO_2_ nanoparticles in the chloroplast [149,150]. Lei et al. [151] hypothesized that the photogenerated electron holes h+ in TiO_2_ nanoparticles capture electrons from water, which accelerates water photolysis and oxygen evolution in PSII.

The effects of ZnO nanoparticles on photosynthesis vary with plant species, the concentration of ZnO nanoparticles, and the growth stage of the plant. For example, Zhao et al. [152] found that the application of ZnO nanoparticles (~25 nm in size) at 800 mg kg^−1^ to maize caused reduced net photosynthesis, stomatal conductance, and chlorophyll content after 20 days of exposure. However, there were no significant effects at 400 mg kg^−1^ at any growth stage. In soybeans, the application of TiO_2_ nanoparticles improved photosynthesis, which could be attributed to the photocatalytic property of TiO_2_ nanoparticles and the enhanced rate of oxygen evolution [153].

The effect of CuO nanoparticles on chlorophyll *a* fluorescence was studied in duckweed (*Lemna gibba* L.) by Perreault et al. [154]. The results showed that CuO nanoparticles decreased the quantum yield of PSII and increased the non-photochemical quenching, suggesting decreased conversion of absorbed light energy via PSII electron transport to form adenosine triphosphate (ATP) and nicotinamide adenine dinucleotide phosphate reduced form (NAPDH_2_). Similarly, Lalau et al. [155] reported that CuO nanoparticles caused a disrupted mitochondrial structure, the dilation of the chloroplast membrane, the distortion of stroma and grana of the chloroplasts, and the alteration of photosynthetic pigments in duckweed species (*Landoltia punctata* L.). In unicellular algae (*Chlamydomonas reinhardtii* L.), the application of CuO nanoparticles coated with polyacrylic acid decreased the PSII electron transport system, which was attributed to the toxicity of copper ions caused by the dissolution of CuO nanoparticles [156].

Gomez-Garay et al. [143] recorded the chlorophyll *a* fluorescence kinetics in 8-month-old moon trefoil (*Medicago arborea* L.) plantlets exposed to 100–400 mg L^−1^ of CeO_2_ nanoparticles. Their results indicated that compared to the control, the application of 100 and 200 mg L^−1^ of CeO_2_ nanoparticles reduced the photochemical efficiency by damaging PSII and the electron transport system. However, Boghossian et al. [157] and Giraldo et al. [105] showed that when the isolated chloroplast from baby spinach leaves was incubated in nano-CeO_2_, the ROS scavenging ability and photosynthetic activity were enhanced. Furthermore, the improved efficiency of photosynthesis in *Arabidopsis* upon CNT exposure was attributed to an increase in the light absorption spectrum and electron transport rates.

### 6.2. Primary and Secondary Metabolism

Primary metabolism includes reactions essential for normal growth, development, and reproduction, including anabolic and catabolic reactions of carbohydrates, proteins, lipids, and nucleic acids. Nitrogen is a key element of proteins, nucleic acids, hormones, alkaloids, etc. Nitrogen is absorbed in nitrate or ammonia by the roots, and NRase is the enzyme involved in converting nitrate to ammonia. Glutamate dehydrogenase and Glutamine synthase are the enzymes involved in converting ammonia to amino acids, subsequently as proteins. The role of nanoparticles in nitrogen metabolism may be associated with the activation or suppression of these enzymes.

Studies have shown that nanoparticles exert both positive and negative effects on protein biosynthesis and degradation, and it depends on the composition, concentration, size, and physicochemical properties of nanoparticles [158]. The exact mechanism of increased protein biosynthesis by nanoparticles is not known. However, studies have shown that nanoparticles can increase the protein content in plants [159,160]. The possible mechanisms associated with protein degradation or denaturation are nanoparticles entering plant cells that can release metal ions that alter protein function. In addition, the specific surface property of nanoparticles can create a layer of the OH^-^ group at the surface. These negative charges will be absorbed into the positively charged side groups of proteins [161].

Yang et al. [93] observed that the application of nano-anatase (25%, 4–6 nm diameter in size) to spinach significantly increased the activities of NRase, GDH, GS, and glutamic-pyruvic transaminase leading to increased nitrate uptake, conversion of inorganic nitrogen into organic nitrogen compounds, and plant biomass compared to control. The exposure of barley to cadmium oxide (CdO) nanoparticles (2–7 nm in size) significantly decreased the contents of primary metabolites like amino acids and saccharides. However, there was no change in the content of secondary metabolites like phenols, Krebs cycle acids, and fatty acids [162]. Krishnaraj et al. [163] found that the application of biologically synthesized Ag nanoparticles (diameter 2–50 nm) induced the biosynthesis of proteins and carbohydrates in waterhyssop (*Bacopa monnieri* L.). Exposure to GO decreased the amino acids and carbohydrate metabolism due to the mitochondrial respiratory dysfunction triggered by GO; however, the ratio of unsaturated to saturated fatty acids increased [164]. Ma et al. [77] observed that the exposure of *Arabidopsis* plants to nano-CeO_2_ and nano-indium oxide (In_2_O_3_) altered the expression of genes involved in sulfur assimilation and the glutathione biosynthesis pathway. Exposure to In_2_O_3_ and CeO_2_ nanoparticles resulted in a ~4- and 2-fold increase in the glutathione synthase transcript, respectively, indicating the nanoparticles’ phytotoxicity effect. The application of a biocompatible hydrated graphene ribbon (0.38 nm in size) increased carbohydrate, amino acid, and fatty acids metabolism, which determined secondary metabolism, nitrogen sequestration, cell membrane integrity, permeability, and oxidation resistance [165].

Research on the effect of nanoparticles on plant secondary metabolites is minimal. Plants produce different secondary metabolites, namely terpenoids, phenols, and alkaloids, in response to environmental conditions. Ghorbanpour and Hadian [166] have reported that under in vitro conditions, MWCNTs enhance the callus induction and total flavonoids in Jamzad (*Satureja khuzestanica* L.), an aromatic plant. Also, the callus extract from Jamzad grown with MWCNTs had a stronger antioxidant activity than butylated hydroxytoluene. A similar increase in total antioxidant activity was observed in sage (*Salvia officinalis* L.), a medicinal plant, by exposing them to TiO_2_ nanoparticles [167].

The changes in secondary metabolites upon MWCNT exposure could be related to altered activities of enzymes like phenylalanine ammonia-lyase, POX, and polyphenol oxidase. Ma et al. [77] showed that *Arabidopsis* plants exposed to nano-CeO_2_ and nano-indium oxide (In_2_O_3_) accumulated higher anthocyanin levels and showed increased expression of the polyphenol oxidase gene associated with the production of anthocyanin. Khodakovskaya et al. [168] reported that carbon-based nanomaterials might activate multiple genes involved in stress signaling pathways similar to plant responses to insects, herbivores, or pathogen attacks. In another study, Zhang et al. [169] showed that Ag nanoparticles enhanced the artemisinin (a sesquiterpene lactone) and ROS content in a sweet wormwood (*Artemisia annua* L.) hairy root culture. The overproduction of ROS in plants by nanoparticle exposure may be another possible mechanism for increased secondary metabolite production. Ghorbanpour [167] evaluated the influence of TiO_2_ nanoparticles on sage, and the result indicated that TiO_2_ improved the total phenol and flavonoid content in leaves compared to the control. Similarly, in Licorice (*Glycyrrhiza glabra* L.), the application of CuO and ZnO nanoparticles increased the phenol and glycyrrhizin (a natural sweetener) content compared to their bulk counterpart [170].

In general, the production of ROS is part of the normal physiological process. It is formed by the inevitable leakage of electrons onto O_2_ from the electron transport chain in the chloroplast, mitochondria, and plasma membranes or as a byproduct of various metabolic pathways localized in different cellular compartments. In other instances such as biotic and abiotic stress, the production of ROS increases, leading to the activation of both enzymatic and non-enzymatic antioxidant defense systems in plants. Several works have reported that nanoparticles tend to induce ROS generation in plants [15,153]. However, reports have also indicated that nanoparticles can scavenge the ROS [25,143,157,171,172]. The level of ROS generation by a nanoparticle depends on the physical and chemical nature of the nanoparticle and the testing systems, namely, the cell types. The most critical physical and chemical structural determinants of nanoparticles that lead to ROS production include size, shape, oxidation status, surface area, bonded surface species, surface coating, solubility, and degree of aggregation and agglomeration. The antioxidant property of the nanoparticle is associated with mimicking the activity of natural antioxidant enzymes like SOD, CAT, and POX [173,174,175].

### 6.3. Understanding the Effects of Nanoparticles in Plants at Omics Level

Nanoparticles can positively influence plant growth, development, and stress tolerance by altering cellular responses due to their smaller size and higher surface area compared to bulk materials. In plants, nanoparticles can modify the growth rate and cell viability at the cellular level through the accumulation of nanoparticles at the plasma membrane, cell wall, chloroplasts, vacuoles, and endoplasmic reticulum. However, the effect of this nanoparticle at the transcriptomic level has not been studied in detail. Several studies have indicated that metallic and non-metallic nanoparticles can induce alterations in gene expression upon prolonged exposure [176]. For instance, SWCNTs increased the expression of seminal root-associated genes like *SLR1* and *RTCS* while decreasing root hair-associated genes like *RTH1* and *RTH3* in maize, indicating that nanoparticles can modify the expression of genes associated with root morphology and function [177]. Similarly, the application of selenium nanoparticles to bitter melon (*Momordica charantia* L.) increased the expression of WRKY1, a transcription factor regulating stress-related genes, as well as *PAL* and *4CL* genes involved in secondary metabolite production [178]. In another study, in *Arabidopsis*, the application of silver nanoparticles @ 5 mg L^−1^ resulted in the upregulation of 286 stress-related genes and the downregulation of 81 genes primarily associated with the pathogen response and hormone signaling [179].

Corn seed treatment with MWCNT increased the expression of water channel proteins, resulting in increased water uptake, higher seed germination, and seedling growth compared to control [180]. Further, *Arabidopsis thaliana* roots treated with TiO_2_ showed the upregulation of 80 genes and the downregulation of 74 genes [181]. All these genes are associated with abiotic and biotic stress responses and signaling. In contrast, the application of CuO nanoparticles to radish (*Raphanus sativus* L.), perennial ryegrass (*Lolium perenne* L.), and annual ryegrass (*Lolium rigidum* L.) resulted in the induction of oxidative damage and mutagenic DNA lesions as evidenced by the accumulation of 7,8-dihydro-8-oxoguanine, 2,6-diamino-4-hydroxy-5-formamidopyrimidine, and 4,6-diamino-5-formamidopyrimidine [182]. These studies showed that nanoparticles can elicit species-specific growth-promoting or phytotoxic effects through the regulation of gene expression [183]. Monitoring gene expression changes offers an effective approach for assessing nanoparticle-induced toxicity, as well as physiological responses.

Proteomics, the study of proteins, involves understanding their structure, function, and interrelationship. It is an effective tool for identifying the quantitative and qualitative changes in protein levels [184]. Understanding how nanomaterials affect plant metabolism using proteomic techniques can explain cell defense, secondary metabolism, and nutrient uptake [185,186,187]. Plants treated with nanoparticles exhibit significant alterations at the proteomic level, with the upregulation and downregulation of various proteins. For example, proteomic analysis of soybean roots treated with silver nanoparticles revealed a decrease in the concentration of proteins related to fermentation, such as pyruvate decarboxylase [185]. In contrast, a study on rice demonstrated an increase in the abundance of proteins involved in cell wall synthesis and cell division [188]. Proteomic analyses of *Brassica napus* subjected to ZnO treatment have demonstrated significant alterations in proteins associated with photosynthesis, carotenoid biosynthesis, and light-harvesting complexes, and these molecular changes were accompanied by a corresponding increase in the photosynthetic rate, as well as increased carotenoid and chlorophyll content, suggesting that ZnO positively influences the photosynthetic performance and pigment synthesis [189].

In addition to their involvement in primary metabolic processes such as photosynthesis and regulatory mechanisms such as signaling, nanomaterials can also alter the expression of stress-associated proteins. Foliar- and soil-applied CeO_2_ significantly affected the proteome of *Phaseolus vulgaris*. Proteins related to stress responses, such as pathogenesis-related proteins and proteins involved in oxidative stress (APX and GPX), were differentially expressed, indicating the role of CeO_2_ nanoparticles in modulating plant stress pathways [190]. The upregulation of stress-related proteins such as SOD and glutathione transferase, which mitigate stripe rust-induced oxidative stress in wheat when treated with TiO_2_ nanoparticles, was reported [191]. Overall, nanomaterials in plant systems significantly altered proteomic profiles, leading to changes in protein expression. These modifications highlight how nanotechnology can optimize physiological processes, boost plant metabolism, and increase stress resilience.

Metabolomics is the study of the quantification of metabolites and the interaction of metabolites produced in plants in response to an external or internal plant condition. Metabolomics is an indispensable platform for understanding post-genomics approaches to discover the networks and interactions of metabolites. Studies have shown that nanoparticles can induce a significant change in metabolite reconfiguration and physiological and genotoxic changes in plants [192,193,194]. The effect of nanomaterials on metabolite adjustment has been studied previously; however, detailed studies are required to understand the effect of nanomaterials on primary and secondary metabolism [195]. Currently, it has been proven beyond doubt that nanoparticles are involved in the overproduction of ROS [196], which can induce oxidative damage, and this was validated by the identification of membrane damage products using gas chromatography and mass spectrometry techniques [197].

Despite the negative effects of nanoparticles on plants, several studies have shown positive responses of plants to nanomaterials, which include increased growth, root growth, and seed yield under normal and stressful environments through adjustments in metabolite production [198]. In rice, the application of TiO_2_ through hydroponics increased hexose phosphate, fatty acids, amino acids, and secondary metabolites [199]. The same nanoparticle in rice applied through soil increased proline and amino acids and decreased the levels of fatty and organic acids [200]. Similarly, the application of CeO_2_ as seed treatment increased lauric and valeric acid and reduced the lignin content in rice [138]. In beans, the foliar application of CeO_2_ with a particle size of 10–30 nm altered terpenes and alkaloids, resulting in changes in terpene hormones [194]. The application of metallic nanoparticles like silver via foliar spray changed the concentration of carnosic acid content in rosemary (*Salvia rosmarinus* L.) [201]. Seed treatment with 10 mg L^−1^ of Ag nanoparticles increased gibberellic acid and decreased cytokinin levels [202]. Silver nanoparticles changed the proportions of sugars and organic acid involved in the seed germination of wheat. Specifically, the levels of soluble sugars and organic acids decreased, resulting in poor growth of the coleoptile and suggesting that silver nanoparticles can affect the sugar metabolism in the endosperm [203]. Overall, it can be concluded that nanoparticles can alter the metabolism of germinating seeds and plants at the vegetative or reproductive stages.

Metallic nanoparticles induce ROS accumulation and reprogram the metabolic pathway, readjusting the levels of metabolites associated with oxidative damage. Similarly, nanoparticles are involved in sugar and organic acid metabolisms, indicating they are directly involved in the primary and secondary metabolism of plants under normal conditions. Studies have explained the role of nanoparticles in alleviating abiotic and biotic stress tolerance. However, the metabolic changes under a combination of nanoparticles and multiple stresses have not been studied in detail and need to be explored to understand the mechanism of action of nanoparticles in plants. The effects of nanoparticles on growth and various physiological processes are presented in Table 1.

## 7. Conclusions

An ecotoxicity assessment of engineered nanoparticles requires the development of proper protocols and guidelines to compare the results of similar types of nanoparticle experiments. Obviously, the nanoparticle uptake processes and movement within the plant system have to be validated using the quantification of content through ICP-MS/MS, localization methods using probes, μ-XRF, and TEM-EDS. Apart from this, information on the accumulation or compartmentalization of nanoparticles in various plant parts needs to be quantified. Furthermore, validation of the effects of nanoparticles’ size, surface coating, surface charge, and plant species on uptake and translocation has to be understood. Knowledge of this will help develop agrochemicals such as nanofertilizers, nanopesticides, nanoherbicides, etc., for precision agriculture.

Similarly, the uptake and translocation of nanoparticles in soil have to be assessed for risk quantification. Life cycle analysis of each nanomaterial should be performed. It is clear that nanomaterials positively or negatively impact plants through antioxidant or pro-oxidant properties, respectively. The pro-oxidant property was associated with nanomaterials’ toxicity. The effects of nanoparticles on the growth, photosynthesis, and primary and secondary metabolism of plants are both positive and negative, indicating that the effect is based on size, charge, concentration, plant species, and stage of growth. To understand the mechanism of action of nanoparticles, the response of nanoparticles at the omics level is necessary. Overall, it is evident that there is scope for the use of nanoparticles in agriculture to achieve food and nutritional security, but the rationale use of nanoparticles without leaving a negative footprint on the ecosystem and/or food system is necessary.

## Figures and Tables

**Table 1:** Influence of nanoparticles on growth and physiological processes in various crop plants.

Type of Nanomaterial	Species	Function	Reference
Seed-based application			
Copper nanoparticles	Turnip (*Brassica rapa* ssp. *rapa*)	Chlorophyll, carotenoid, and sugar content decreased, while proline and anthocyanins were enhanced. Also, plants showed increased oxidative damage.	[204]
	Rice (*Oryza sativa* L.)	Germination rate, root and shoot length, biomass, photosynthetic rate, transpiration rate, stomatal conductance, maximal quantum yield of PSII photochemistry, and photosynthetic pigment contents were reduced. In addition, the accumulation of Cu was observed in chloroplasts.	[205]
Gold nanoparticles	Soybean (*Glycine max* L.)	Gold nanoparticles translocated and accumulated in plants after seed inoculation. Also, induced quenching of chlorophyll *a* fluorescence, and the quenching depends on the particle size and concentration. The quenching property was associated with photoinduced electron transfer from excited chlorophyll molecules to gold nanoparticles.	[206]
	Cowpea [*Vigna unguiculata* (L.) Walp]	No effect on growth, proline, and malondialdehyde content. Cowpea possessed the potential to withstand Au^3+^ stress as the phenolics released by the seed coat of germinating seeds had the potential to reduce toxic Au^3+^ to form non/less toxic Au nanoparticles.	[207]
Iron-based nanoparticles	Rice	Nanoprimed plants had broader leaves, higher growth, biomass, and tiller number.	[208]
		Increased endogenous ROS via Fenton’s reaction, and upregulated OsPIP1 gene leading to higher relative water contents.	[209]
Zinc-based nanomaterials	Brassica (*Brassica oleracea* var *italic*)	Increased seedling growth and seed germination. Along with this, increased proline, phenols, and sugar content were observed.	[210]
	Groundnut (*Arachis hypogea* L.)	Improved seed germination and seedling vigor, resulting in a higher pod yield.	[111]
Cerium-based nanomaterials	Rice	Enhanced electrolyte leakage and lipid peroxidation were observed at 500 mg L^−1^. Also, concentration-dependent changes in oxidative stress and antioxidant defense systems were observed.	[138]
	Pearl millet [*Pennisetum glaucum* (L.) R. Br.]	Decreased downy mildew incidence and increased expression of resistance genes were observed.	[211]
Soil application			
Copper nanoparticles	Wheat (*Triticum aestivum* L.)	Impaired root and shoot growth up to 500 mg kg^−1^ of soil.	[212]
	Wheat	The number of grains per spike and 1000 grain weight was increased. In addition, enhanced expression of proteins associated with glycolysis and starch degradation and antioxidant enzyme activity was increased.	[213]
	Sweet potato (*Ipomoea batatas* L.)	Cu reduced biomass yield. Cu accumulated more in peels than in the tuber flesh.	[214]
Gold nanoparticles	Tomato (*Solanum lycopersicum* L.)	Induces growth of tomato plants and did not have any negative impact on plant growth.	[215]
Titanium nanoparticles	Soybean	Present in roots; no effects on plant growth, nutrient content, or the composition of root-associated microbiota.	[216]
Iron-based nanomaterials	Rice	Addition of 100 mg nZVI per kg soil containing 100 mg of pentachlorophenol increased grain yield and decreased grain pentachlorophenol content.	[217]
	Maize (*Zea mays* L.)	Iron was accumulated in the roots and translocated to leaves. At 50 mg kg^−1^, root elongation was enhanced. At 500 mg kg^−1^, pigments, lipid peroxidation, and polyphenolic levels in leaves were increased.	[218]
	Groundnut	Increased root length, plant height, biomass, and chlorophyll content. Also, altered the phytohormone contents and antioxidant enzyme activity.	[219]
Zinc-based nanomaterials	Cucumber (*Cucumus sativus* L.)	Altered carbohydrate fractions, but no changes in protein content.	[220]
	Tomato	Decreased root growth, shoot growth, pigment concentration, photosynthetic efficiency, and chlorophyll fluorescence parameters in a concentration-dependent manner.	[221]
	Pearl millet	Improved growth and yield.	[122]
	Wheat	Under drought, the average time to panicle initiation was reduced by 5 days, and grain yield was increased.	[222]
	Maize	Increased the grain yield	[223]
Cerium-based nanomaterials	Maize	Oxidative damage in the phloem, xylem, bundle sheath cells, and epidermal cells of shoots was observed at 800 mg kg^−1^, along with increased oxidant concentration and antioxidant enzyme activity was decreased over control.	[31]
	Wheat	First-generation exposure had a significant impact on the second-generation wheat plants on growth, yield, and nutrient content.	[224]
	Soybean	Altered nutrient composition of seed.	[225]
	Coriander (*Coriandrum sativum*)	Altered the quality, especially carbohydrate fractions.	[226]
Selenium nanoparticles	Eggplant (*Solanum melongena* L.)	Increased leaf area and high-temperature stress tolerance.	[227]
Carbon-based nanomaterials	Soybean	C_60_ induced phytotoxicity and reduced the biomass and growth.	[121]
	Tomato	Increased number of flowers and fruit.	[228]
Foliar application			
Gold nanoparticles	Black mustard (*Brassica nigra* L.)	At 10 ppm, increased stem diameter, number of leaves and branches per plant, total chlorophyll, grain yield, oil content, and seed yield. Apart from this, reduced membrane damage and ROS levels were observed.	[229]
	Soybean	Improved the growth under flooding stress.	[185]
Iron-based nanomaterials	Tomato	Increased plant height, leaf number and area, shoot and root fresh and dry weights, and chlorophyll content. Also, photosynthesis, stomatal conductance and transpiration rates, total yield, and yield components were increased.	[230]
	Wheat	Increased photosynthetic pigments and total phenolic contents.	[231]
	Wheat	Increased spike weight, 1000 grain weight, biological yield, grain yield, and protein content were observed.	[232]
	Wheat	Increased chlorophyll content, antioxidant enzyme activity, harvest index, and 1000-grain weight were observed.	[233]
Cerium nanomaterials	Rice	Increased antioxidant contents in shoots and roots up to 500 mg L^−1^. Above that, oxidative damage was observed.	[138]
	Maize	No translocation within the plant system and was not dependent on open or closed stomata.	[13]
	Greengram	Improved drought tolerance by increased photosynthetic rate, chlorophyll content, biomass, and antioxidant defense system.	[234]
	Sorghum	Improved drought stress tolerance by ROS scavenging.	[235]
	Tomato	Inhibited growth by causing oxidative stress and bioaccumulation is dose-dependent.	[236]
Selenium nanoparticles	Tomato	Increased root and shoot biomass, fruit production, and post-harvest shelf-life	[237]
	Cucumber	Se application alleviates salinity and high-temperature stress and increases cucumber yield. Foliar Se application promoted biostimulant effect and increased cucumber plant biomass.	[238]
Carbon-based nanomaterials	Coriander	Increased plant height, branch number per plant, total dry weight, number of inflorescences per coriander plant, fruit yield per plant	[239]
	Maize	Nanochitosan decreased photosynthetic rate; later it increased photosynthetic rate, stomatal conductance, and transpiration rate.	[240]
	Maize	Improved the antioxidant defense enzymes and sucrose translocation	[241]
Soil and foliar application			
Titanium nanoparticles	Tomato	Increased the light absorption and chlorophyll content.	[242]
Iron-based nanoparticle	Soybean	Positive effects on root elongation, shoot weight, leaf area, and chlorophyll index were observed.	[243]
Zinc-based nanoparticle	Tomato	Aerosol-mediated application was found to be more effective than soil-mediated application, as revealed by ZnO uptake.	[242]
	Soybean	Increased shoot N, K, Zn, B, and Cu content.	[244]
Hydroponics and solution-based application			
Copper nanoparticles	Maize	Transported from roots to shoots via the xylem and translocated from shoot to root via the phloem, indicating its mobility pathway.	[73]
	Lettuce (*Lactuca sativa*)	Reduced the root length, P, and Fe and increased antioxidant enzyme activity.	[245]
Gold nanoparticles	Barley (*Hordeum vulgare* L.)	Au nanoparticles did not enter the root, regardless of their size and concentration. If applied directly into the cells of a root, do not move into neighboring cells.	[246]
Titanium nanoparticles	Moldavian balm (*Dracoce phalummoldavica* L.)	Increased salinity tolerance through increased antioxidant enzyme activity.	[247]
Iron-based nanomaterials	Rice	Low doses of ZVI and Fe_3_O_4_ NPs promoted growth through antioxidant activity under Fe-deficient conditions.	[248]
Zinc-based nanoparticle	Tomato	Zn accumulation in roots and shoots followed the order of Zn^2+^ ions > nZn > nZnO > bulk ZnO. Higher concentration (200 mg Zn L^−1^) induces oxidative stress.	[249]
Cerium-based nanomaterials	Cotton	Decreased indole-3-acetic acid and abscisic acid concentrations in the roots.	[109]
	Cucumber	Biotransformation of nanoceria within the plant system. Ce presented in the roots as CeO_2_ and CePO_4_, while in the shoots as CeO_2_ and cerium carboxylates.	[250]
	Arabidopsis	Increased the expression of glutathione synthase enzyme.	[76]
Carbon-based Nanomaterials	Soybean	Induced very little or no effect on root and shoot growth.	[251]
	Soybean	Increased photosynthetic rate.	[252]
	Arabidopsis	SWCNT transported within the lipid envelope of chloroplasts and increased the photosynthetic rate.	[105]
Tissue culture and agar-based application			
Copper nanoparticles	Arabidopsis	Reduced biomass, total chlorophyll, and anthocyanin and increased lipid peroxidation and proline contents.	[96]
	Indian mustard (*Brassica juncea* L.)	Decreased shoot growth and reduction in total chlorophyll and carotenoid contents.	[96]
	Greengram [*Vigna radiata* (L.) R. Wilczek]	Decreased shoot and root length, biomass, and total chlorophyll content. Increased ROS content in roots and lipid peroxidation were observed.	[253]
Gold nanoparticles	Arabidopsis	A positive correlation between the expression of microRNAs and seed germination, growth, and antioxidant potential was observed.	[254]
Zinc-based nanomaterials	Green gram	Seedling growth was increased up to 1 mg L^−1^; after that toxic effect was noticed.	[255]
	Chickpea (*Cicer arietinum*)	Seedling growth was increased up to 20 mg L^−1^; after that toxic effect was noticed.	[255]
Cerium-based nanomaterials	Green microalga (*Pseudokirchneriella subcapitata*)	The toxicity is associated with the percentage of Ce^3+^ ions at the surface of the nanoparticles and oxidative stress. Also, algae did not internalize the nanoceria like animal and human cells.	[256]
Carbon-based nanomaterials	Barley/Soybean/Maize	Increases biomass production, root length, and water channel proteins in plants.	[117]
	Tobacco (*Nicotiana tobacum* (BY-2) cells)	Carbon nanotubes can traverse across both the plant cell wall and cell membrane.	[257]
	Tobacco callus culture	Upregulated genes involved in cell division/cell wall formation and water transport.	[81]
	Chilli (*Capsicum annuum* L.)	The supplementations of culture media led to changes in the root architecture and differentiation.	[258]

## Data Availability

The study did not report any data.
